# Novel Delivery Systems of Polyphenols and Their Potential Health Benefits

**DOI:** 10.3390/ph14100946

**Published:** 2021-09-22

**Authors:** Bianca Enaru, Sonia Socaci, Anca Farcas, Carmen Socaciu, Corina Danciu, Andreea Stanila, Zorita Diaconeasa

**Affiliations:** 1 Department of Food Science and Technology, Faculty of Food Science and Technology, University of Agricultural Science and Veterinary Medicine, 400372 Cluj-Napoca, Romania; bianca.enaru@stud.ubbcluj.ro (B.E.); sonia.socaci@usamvcluj.ro (S.S.); anca.farcas@usamvcluj.ro (A.F.); carmen.socaciu@usamvcluj.ro (C.S.); 2 Department of Pharmacognosy, “Victor Babes” University of Medicine and Pharmacy Timisoara, 300041 Timisoara, Romania; corina.danciu@umft.ro

**Keywords:** polyphenols, liposomes, cancer therapy, chemoprevention, health

## Abstract

Liposome-based delivery systems have been studied and used more frequently in recent years due to their advantages, such as low toxicity, specificity, and the ability to protect the encapsulated substance from environmental factors, which could otherwise degrade the active compound and reduce its effectiveness. Given these benefits, many researchers have encapsulated polyphenols in liposomes, thus increasing their bioavailability and stability. Similarly, polyphenols encapsulated in liposomes are known to produce more substantial effects on targeted cells than unencapsulated polyphenols, while having minimal cytotoxicity in healthy cells. Although polyphenols play a role in preventing many types of disease and generally have beneficial effects on health, we solely focused on their chemopreventive effects on cancer through liposomes in this review. Our goal was to summarize the applicability and efficacy of liposomes encapsulated with different classes of polyphenols on several types of cancer, thus opening the opportunity for future studies based on these drug delivery systems.

## 1. Introduction

**Figure 1:**
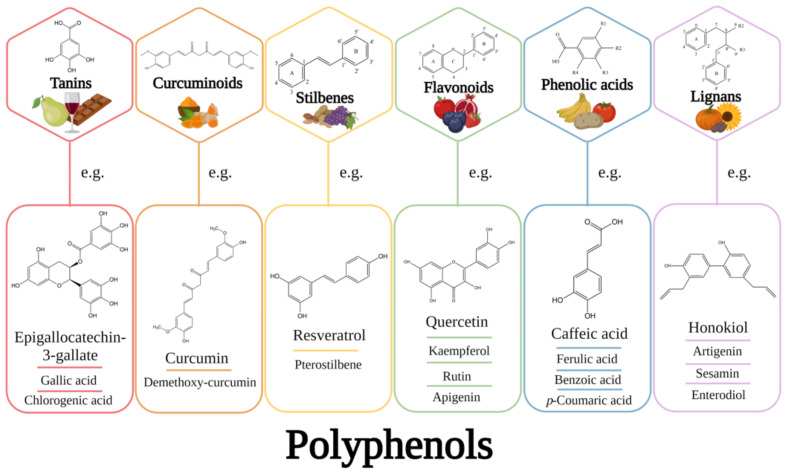
Classification of polyphenols and their biological sources.

Polyphenols are a large group of secondary metabolites, consisting of one or more aromatic rings to which one or more hydroxyl groups are attached [1]. They are found in large quantities in various foods, such as fruits, vegetables, coffee, tea, chocolate, and wine, as illustrated in Figure 1 [2]. Depending on their origin, biological function, and chemical structure, polyphenols can be classified into two different categories: flavonoids and non-flavonoids. The first class comprises flavonols, flavones, flavanones, anthocyanidins, catechins, isoflavones, and chalcones. On the other hand, the second class comprises phenolic acids (such as hydroxybenzoic acid and derivates, along with hydroxycinnamic acids and derivates) and others, including stilbenes, lignans, curcuminoids, and tannins. It is worth mentioning that, among all known polyphenols, 60% belong to the flavonoids group, and 30% include phenolic acids [3].

In addition, the industry is particularly interested in the extraction and characterization of polyphenols from natural sources to employ their potential bioactive properties. Polyphenolic substances such as phenolic acids, flavonoids, anthocyanins, and tannins can all be used as biomarkers for chemotaxonomic classification or fruit quality markers because of their potential health advantages [4].

The purpose of this research is to provide an overview of polyphenols encapsulated in liposomes and their beneficial effects on health, more specifically in anticancer therapy. Taking into account the numerous studies conducted in recent years, in which different types of liposomes were encapsulated with polyphenols and used on multiple cancer lines, we wanted to summarize them schematically as a function of their class. Thus, we review the most important polyphenols in each class, outline the effect produced in their free form, and then describe the effects produced by their liposomal formulations. In this way, we open the opportunity for future studies based on these drug delivery systems, which are the future in cancer therapy.

The literature search was done using the PubMed, Web of Science, and Scopus databases, as well as the academic search engine Google Scholar. The following keywords were used: polyphenols* AND liposomes, polyphenols* AND stability, polyphenols* AND cancer, polyphenols* AND potential health benefits* AND chemoprevention. The results were screened on the basis of their titles, abstracts, and full-text availability. All non-English publications were excluded from the present review. Filter limits (such as text availability, article type, and publication date) were not applied. The time window was up to 1 July 2021.

## 2. The Need to Encapsulate Polyphenols in Liposomes

Polyphenols have recently come to the attention of researchers from various fields such as pharmaceuticals, cosmetics, and food, due to their potential for prevention and protection against numerous diseases and their beneficial properties on an individual’s health. Many beneficial effects of polyphenols are attributed to their antioxidant, anti-inflammatory, antimicrobial, antimutagenic, anticarcinogenic, and digestion-stimulating properties [5]. Over the years, the effects of polyphenols have been intensively studied in preventing a wide range of conditions, such as diabetes, obesity, cardiovascular disease, neurodegenerative disorders, and cancer [6].

In everyday life, most people pursue a diet rich in polyphenols sourcing from various foods that contain them, such as green vegetables, fruits, soybeans, tea, beer, coffee, or red wine. However, there may be variations in their quantities consumed from one country to another in terms of their consumption. For example, in the United States, Spain, and Australia, estimated consumptions of flavonoids are about 190, 313, and 454 mg/day, respectively [7]. However, because foods contain a variable amount of flavonoids due to their growing environment, storage, processing, or cooking, such data are generally considered approximations of food contents [8].

Because vegetables, fruits, coffee, red wine, and tea are all rich in polyphenols, current research focuses on identifying those responsible for a particular pharmacological or chemopreventive effect, making considerable efforts to elucidate molecular mechanisms of action [9].

**Figure 2:**
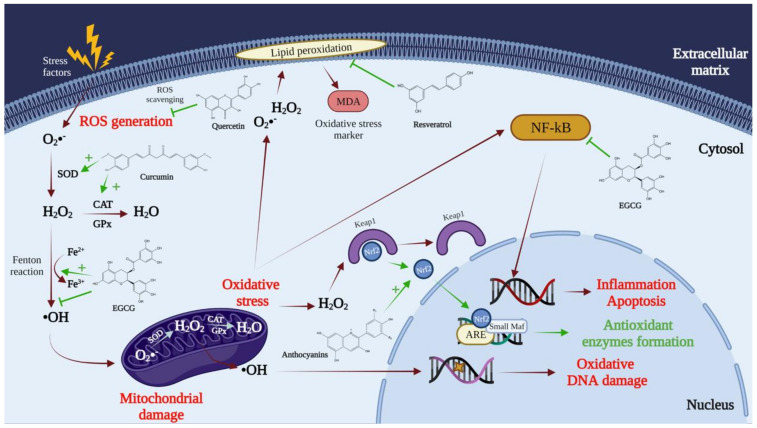
The mechanisms via which polyphenols act on free radicals, reducing oxidative stress. SOD, superoxide dismutase; CAT, catalase; GPx, glutathione peroxidase; MDA, malondialdehyde; Nrf2, nuclear factor erythroid 2-related factor 2; ARE, antioxidant response elements; Small Maf, musculoaponeurotic fibrosarcoma proteins; Keap1, Kelch-like ECH-associated protein 1; NF-kB, nuclear factor kappa-light-chain-enhancer of activated B cells.

Most of the pathological conditions mentioned are related to oxidative stress caused by reactive oxygen and nitrogen species (Figure 2). Thus, in particular, polyphenols represent the primary antioxidant agent in fruits, with superior efficacy to vitamin C [10]. It has been determined that the substances with the most substantial antioxidant effect, which can neutralize free radicals by donating an electron or a hydrogen atom, are polyphenols. Therefore, polyphenols inhibit the generation of free radicals while reducing the oxidation rate by inhibiting the formation of active species and free-radical precursors or by deactivating them. Polyphenols usually perform as direct radical eliminators in lipid peroxidation chain reactions (chain breakers). They transfer an electron to the free radical, neutralizing it; after that, they become stable radicals (less reactive), and the chain reactions terminate [11]. In addition to the functions presented above, polyphenols also act as metal chelators. They can cause the chelation of transition metals such as Fe^2+^, and this process will directly reduce the rate of the Fenton reaction and prevent oxidation caused by highly reactive hydroxyl radicals [12,13]. It is also known that polyphenols can act as co-antioxidants and are part of the regeneration process of essential vitamins [14]. These compounds can induce antioxidant enzymes such as glutathione peroxidase, superoxide dismutase, and catalase that decompose hydroperoxides, hydrogen peroxide, and superoxide anions. In contrast, they can also inhibit the expression of enzymes such as xanthine oxidase [15].

Through the mechanisms presented above, polyphenols are involved in critical activities that reduce oxidative stress. However, their roles at the cellular level are believed to be much more complicated, requiring further studies to elucidate them. Some polyphenols such as flavonoids are absorbed in the gastrointestinal tract, but their plasma concentration is low (1 µmol/L). The leading cause of this is the rapid metabolism of human tissues [11].

According to research on polyphenols, they have been shown to have the capacity to diminish inflammation by inhibiting edema, suppress the development of tumors, possess proapoptotic and anti-angiogenic properties, modulate the immune system, improve capillary resistance by acting on blood vessel components, protect the cardiovascular system, and limit weight gain [16].

Even though polyphenols have health benefits, the amount of polyphenols that can be administered orally is not enough to reach the concentration needed for systemic therapies to be effective. Characteristics such as low water solubility, poor absorption, and rapid metabolism play a role in decreasing the oral bioavailability of polyphenols [9]. Although there are several definitions of the term bioavailability, the best of these expressions is the part of an ingested compound that can reach the systemic circulation and be distributed to the various targeted tissues where it can exert its biological action [17]. Thus, only a small amount of the molecules administered orally are absorbed due to insufficient gastric residence time, low permeability, or low solubility. The absorption of polyphenols from food is always influenced by their chemical structure (degree of glycosylation or acylation), ability to conjugate with other phenols, molecular size, degree of polymerization, and water solubility. Therefore, the bioavailability of these compounds is low when they are administered orally, due to their poor solubility and rapid metabolism (polyphenols are metabolized extensively in tissues and by the colonic microbiota), as well as their membrane permeability and incompatibility with a process of passive diffusion [18].

Another disadvantage of polyphenols is that they are very unstable and sensitive to environmental factors such as temperature, light, oxygen, acidic pH, and the enzymatic activity in the digestive system. These characteristics can decrease the concentration of polyphenols and even cause a total or partial loss of bioactivity [2]. Thus, in order to have an efficient oral bioavailability, the hydrophilic part of the natural compound (which ensures its dissolution in the gastrointestinal fluids) must be in a balance with the lipophilic part (which has a role in crossing the lipid biomembranes) [19].

Over the years, attempts have been made to remedy these deficiencies by using various drug delivery systems to improve their bioavailability and therapeutic efficacy [9]. Among the approaches that were evaluated, formulation with cyclodextrins [20], simple emulsions, gels, and lipid nanocapsules [21], nanoemulsion [22], or liposomes [23,24] can be listed. Thus, researchers have managed to produce liposomes in which both hydrophilic and lipophilic substances can be incorporated, with high encapsulation efficiency and controlled drug release [25]. An advantage of these compounds is their ability to change the membrane’s fluidity, thus achieving easier distribution of plant extracts to the target site. At the same time, the extract has a soluble character, thereby readily determining its location in the structure of liposomes since hydrophilic extracts are encapsulated in the aqueous phase, and amphiphilic and lipophilic compounds are found in the lipid layer of the liposome to reduce a material loss [26,27].

In addition to studies based on polyphenols as therapeutic agents in new therapies and with the development of drug delivery systems, the role of these compounds extends beyond therapeutic agents. They can also be used as primary component modules in drug delivery systems that are innovative. Polyphenols are not only incorporated in drug delivery systems to treat various illnesses, but also frequently employed as fundamental components of novel drug delivery systems due to their significant biological activity. Thus, considering the low toxicity and availability of natural substances, the production of novel drug delivery systems takes advantage of the physical and chemical characteristics of naturally active substances to achieve the design and assembly of drug delivery systems [28].

Polyphenols’ intrinsic amphiphilic characteristic is, thus, critical for their use as functional components in novel drug delivery systems. Specifically, the hydroxy groups of polyphenols (as a hydrophilic component) are responsible for this, acting as donors or acceptors of hydrogen bonds, thus playing a role in the interactions between polyphenols and a wide variety of bioactive substances or carriers. In contrast, the aromatic benzene unit of polyphenols (as the hydrophobic component) allows other materials to be hydrophobic, which aids in the development of drug delivery methods [28].

Therefore, due to the chemical structure of polyphenols, they can be combined with a wide variety of materials, such as metals, proteins, polymers, and small molecular compounds through hydrogen bonds, covalent bonds, metal coordination bonding, π–π stacking, and hydrophobic and electrostatic interactions. Polyphenols can not only successfully facilitate drug loading and delivery when combined with other bioactive materials or small molecules, but also safeguard the effective components and nanostructure of drug delivery systems [28].

## 3. What Is a Liposome?

**Figure 3:**
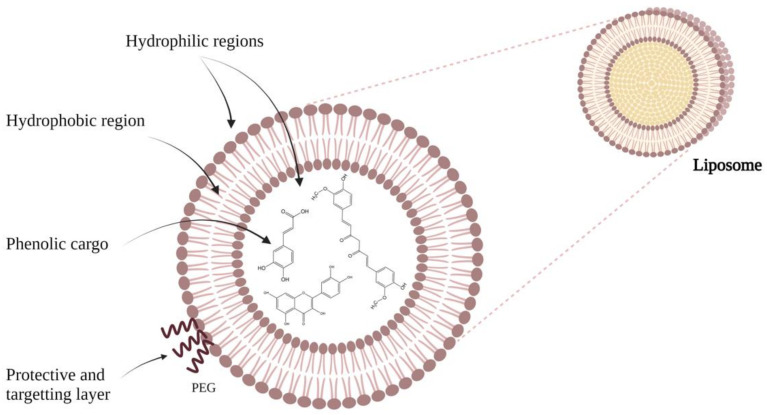
Schematic representation of liposomes.

Liposomes were first described as swollen phospholipid systems in 1965 at Cambridge University by Bangham and coworkers [29], while testing the institute’s new electron microscope by applying negative dye to dry phospholipids [30,31]. The main mechanism for obtaining them is via the self-assembly of surfactants and natural/synthetic lipids in an aqueous solution [32]. From a chemical point of view, liposomes are spherical phospholipid vesicles, nanoscopic or microscopic, composed of one or more concentric lipid bilayers, which encompass an aqueous core (Figure 3). They are generally made up of phospholipids and cholesterol, making them readily biodegradable [33]. Thus, the phospholipids used can be natural, such as phosphatidylcholine, phosphatidylethanolamine, and phosphatidylserine or obtained by synthesis such as disloyal phosphatidylcholine, destroy phosphatidylcholine, and disloyal phosphatidylethanolamine. After the phospholipid membrane is formed, cholesterol can be incorporated at a high concentration [34].

A considerable advantage of liposomes is their flexibility, as they can have different compositions, dimensions, and characteristics of drug release. Liposomes are specially designed to be multifunctional, and they have different components that can control specific characteristics, such as half-life, permeability, biodistribution, and specificity [35]. Due to their low toxicity, and their ability to protect the encapsulated substances from the environmental factors of the target location, they are preferred to incorporate various compounds, including antibiotics, peptides, and polyphenols, as reviewed in [36].

Although there are multiple benefits of using liposomes in drug development, these delivery systems also have some major disadvantages, for which solutions are still being sought. The most common shortcoming is their low in vivo stability, mainly due to lipid oxidation and hydrolysis, or fission and fusion processes. At the same time, the encapsulated hydrophilic substance can leak or even be lost before reaching the target site. Numerous studies are being done to eliminate these disadvantages. It has been concluded that these problems can be avoided if the composition of liposomes is modified via the addition of adjuvants and antioxidants or via post-preparation processes [37]. Coating liposomes with biopolymers such as chitosan, a nontoxic, biodegradable, and indigestible polysaccharide, is another approach to increase their stability [38]. Thus, using this method has led to improved results in terms of the stability of liposomes during storage, their degradation, and their protection in case of changes in environmental conditions. Furthermore, the coating also enlarges the entrapment efficiency, most probably due to the interactions of polyphenols with chitosan [39].

Various studies have reported that the encapsulation of substances in liposomes can alter their physical properties. The chemical nature and the amount of the encapsulated compounds have an important role in liposomal formulation development.

A large amount of charged carotenoids, for example, will cause oxygen to enter the membrane and lead to instability of the liposomal membrane [40].

## 4. Applications of Liposomes

As outlined above, liposomes are delivery systems that have the ability to encapsulate and release controlled different types of substances, including drugs, nutraceuticals, and even genes, making their use extremely wide [41,42]. Initially, the applications of liposomes were restricted in the medical field, before being later used in cosmetics. However, over time, their popularity has been expanded to other areas, such as delivering vaccines, hormones, enzymes, and vitamins in the body [43]. Liposomes show great flexibility as they can be injected intravenously, intramuscularly, or subcutaneously (liquid suspensions); furthermore, they can be inhaled (aerosol of liposome suspension or lyophilized powder), applied directly to the skin as a suspension, cream, or gel, or even ingested (any of the physical forms) [44].

Considering the benefits offered by liposomes, involving the possibility of large-scale production, the natural ingredients (such as eggs, milk, and soy) involved in manufacturing, biocompatibility, and the ability to transport a wide range of bioactive compounds, they are currently used in many areas, as overviewed in this section [43].

The first popular topic of discussion is drug targeting, allowing to enhance the specificity of a drug that targets the desired cell/tissue. Liposomes can be encapsulated with opsonin and ligands (containing antibodies, apoproteins, hormones). The ligand will specifically recognize the receptor sites and determine the direction of the liposomes to those target sites, where they will accumulate and achieve the anticipated effect. By doing so, liposomes will not be recognized and eliminated by the reticuloendothelial system (liver, spleen, and bone marrow), while the toxicity produced by drugs in untargeted cells/tissues will be minimized [45].

Due to their basic characteristics, namely, the ability to encapsulate various biological substances that can then be delivered to epidermal cells, liposomes are also used in the pharmaceutical and cosmetic fields, e.g., dermatology. Skin hydration is the most critical aspect in skincare. Accordingly, most applications in the field of cosmetics are concerned with this issue of balancing the moisture of the skin. Liposomes can simply hydrate the skin, thus reducing skin dryness, which is the main factor causing skin aging. In addition to this first applicability of liposomes in cosmetics, they can also encapsulate anti-inflammatory agents, immunostimulants, and enhancers of molecular and cellular detoxification, which can produce some therapeutic effects on several skin problems such as dark circles, wrinkles, and age spots [41].

Likewise, due to the rapid development of the food industry in recent years, adding functional compounds to food products has gained more attention. Thus, functional compounds that help to control the flavor, color, texture, or preservative properties of food products have been more widely employed in liposomes. However, these functional compounds are sensitive to environmental factors, processing, and conditions in the gastrointestinal tract, whereas encapsulation could remedy these inconveniences, making liposomes the suitable candidates in such cases [43].

Although there are drawbacks to the degree of encapsulation of polyphenols in liposomes, several studies have reported that both the bioavailability and the efficacy of polyphenols encapsulated in liposomes are improved compared to the free active substance and other transport systems. Various formulations of liposomes showed improved results in terms of the solubility of many polyphenols, for example, resveratrol, quercetin, curcumin, and puerarin [9], as detailed in Table 1.

According to Table 1, the encapsulation ratio varies, even in the case of the same encapsulated polyphenol, and it can be concluded that the encapsulation rate is dependent on the structure of the polyphenol, the lipid composition of the liposome, and its formulation [9].

The liposome–polyphenol complex has another crucial benefit, which is its increased chemical stability, thereby maintaining long-term efficacy [9] or inducing effects that cannot be achieved otherwise by administering free polyphenols [60]. At the same time, the active polyphenols can be injected into nonorganic solvents due to the improved solubility of the substance in the liposomes, which decreases the systemic toxicity and increases the maximum dose tolerated by the body, thus enabling administration of a higher amount of polyphenols in vivo [9].

**Figure 4:**
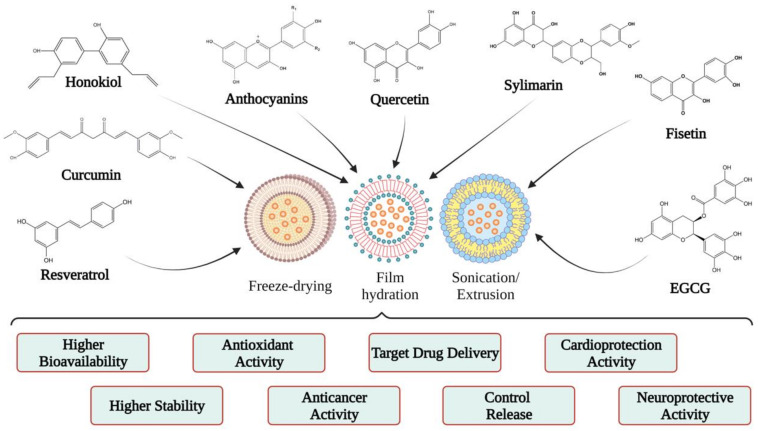
Examples of liposomal forms developed for polyphenol biological studies.

Studies on the use of polyphenols encapsulated in liposomes performed in vitro/in vivo have revealed that they have similar or better efficacy than free polyphenols [46,63]. Therefore, starting from this idea, several researchers have encapsulated different polyphenols in liposomes to observe the effects and benefits of each, a topic that is discussed in the next section (Figure 4).

According to all available data in this field, it is now obvious that the lipid composition of liposomes exhibits excellent flexibility and can be adapted to the substance of interest. Their surface can be modified in such a way that they can stay longer in circulation and acquire better targeting properties [69]. By changing the membrane’s charge via incorporating antibodies or immunoglobulins, the liposomes will have a much higher specific cellular affinity. Liposomes can also be produced to respond to a certain pH, temperature, or a specific organ [70]. Although Ali et al. (2013) investigated the link between lipid geometry in liposomes and the encapsulation of insoluble substances, more studies in this direction are required to determine and quantify the effectiveness of incorporating drugs into lipid layers [71].

## 5. Polyphenols Encapsulated into Liposomes and Their Potential Health Benefits

### 5.1. Quercetin

Quercetin (QC) is a flavonoid plant coming from the word “quercetum” (oak forest), which is part of the Fagaceae family and genus *Quercus*. The literature on QC has highlighted several important characteristics such as its antioxidant, anticarcinogenic, antiviral, anti-obesity, anti-inflammatory, and antihypertensive activities [72]. Due to its antioxidant character, quercetin can eliminate free radicals [73]. This property originates from a large number of conjugated hydroxyl and orbital groups through which QC can donate electrons and hydrogen or eliminate H_2_O_2_ and superoxide anions.

According to existing data, QC has shown anticancer effects on several mechanisms. For example, it has been shown that QC caused cell-cycle arrest in the G2/M phase by activating the p53 tumor suppressor protein, thereby inhibiting the activity of cyclin dependent kinase 2 (CDK2), cyclin A, and cyclin B [74]. In addition, this flavonoid can suppress the synthesis and expression of heat-shock protein and block the signal transduction pathways by inhibiting protein tyrosine kinase and downregulating oncogene expression (c-myc, ki-ras) [75]. Regarding the action of quercetin in angiogenesis, it has been shown that it affects the VEGFR-2 mediated pathway, causing under-expression of the AKT (protein kinase B) regulatory factor, thus inhibiting blood vessel growth and restricting tumor growth in prostate and breast cancer [76]. Quercetin can also determinate apoptosis in tumor cells by stimulating proapoptotic proteins such as BAX and caspases (3, 6, 7, 8, and 9). Furthermore, QC can stop the expression of antiapoptotic proteins such as Bcl-2 and terminate cancer metastasis. In order to form metastases, epithelial-to-mesenchymal transition (EMT), a process that involves downregulation of epithelial-type proteins (e.g., E-cadherin), and stimulation of expression of mesenchymal markers, including N-cadherin and Vimentin, must occur. Thus, in this case, quercetin can decrease the occurrence of EMT by overexpressing E-cadherin and under-expressing N-cadherin and Vimentin [76].

**Figure 5:**
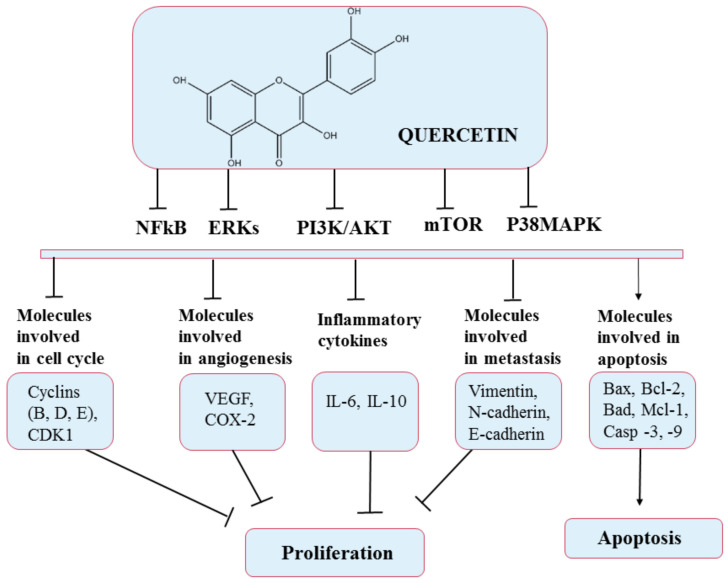
Chemical structure of quercetin and mechanism of action of quercetin through different molecular targets resulting in apoptosis or stopping proliferation.

The most recent investigation reported that QC could inhibit the growth of different types of cancer cells, including colorectal, prostate, liver, pancreatic, breast, kidney, lung, and ovarian, via modulation of various cellular processes (Figure 5). In addition, QC can exhibit selective cytotoxic activity toward cancer cells without producing adverse effects on normal cells [77]. On the other hand, QC has slight aqueous solubility and bioavailability and is rapidly metabolized, and these disadvantages can diminish its effectiveness in treating diseases [78].

Given both the advantages and the disadvantages of this compound, its encapsulation in liposomes was considered to increase its effectiveness. Below, we summarize a study conducted by Saraswat and Maher in 2020. In this study, the authors encapsulated QC in stealth liposomes to observe the effects of the concentration of PEGylated lipids (polyethylene glycosylated) on the liposomal physicochemical parameters and on their stability, critical to deliver the compound efficiently in vitro [72]. For this purpose, the authors used QC (≥95% HPLC), produced stealth liposomes via the traditional method (thin-film hydration), and tested the liposomes encapsulated with QC on cervical cancer cells (HeLa). The authors used QC concentrations of 5–200 μM, and each was left to act for 24, 48, and 72 h. Simultaneously, the cytotoxicity of this type of liposome was evaluated using the same cell line, HeLa. Additionally, various concentrations of QC were encapsulated in the liposomes, measuring the cytotoxic effect at 24, 48, and 72 h, respectively. Liposomes without encapsulated substance were used as a control, and their cytotoxic effect was determined under similar conditions. Lastly, the IC_50_ values of the stealth liposomes encapsulated with QC were calculated. According to their results, both free QC and QC encapsulated in liposomes managed to inhibit cell growth in a time-dependent manner, producing a maximum cytotoxic effect at 72 h intervals. However, it should be noted that liposomes encapsulated with QC had a significantly higher cytotoxic effect on the HeLa cell line compared to freely administered QC at all three time intervals. The IC_50_ value obtained at 24 h for free QC was higher than 200 μM compared to QC-loaded liposomes (184 μM). The IC_50_ value of QC (134 μM) achieved at 48 h was also higher than that of QC-loaded liposomes (40 μM), a trend which continued at 72 h (free QC, 65 μM; QC-loaded liposomes, 14 μM). Overall, the cytotoxicity determined by QC-loaded liposomes was more prominent compared to QC alone, considering the statistically significant difference obtained in their IC_50_ values. In addition to the cytotoxic effect obtained, chemopreventive effects were also observed at concentrations lower than 5 μM (most likely determined by the antioxidant activity of QC) [79,80]. Instead, at high QC concentrations, apoptosis could be observed in the HeLa cell line due to reactive oxygen species (ROS) generation, as well as activation of p53 protein and caspases [81].

On the other hand, Long and colleagues assessed the properties of quercetin encapsulated in PEGylated liposomes using cisplatin-resistant (A2780cp) and cisplatin-sensitive (A2789s) human ovarian cancer models. This study took place both in vivo and in vitro, and the goal was to observe the anticancer effects of this polyphenol. The results demonstrated that liposomes with quercetin caused cell-cycle arrest, induced apoptosis, and inhibited cell proliferation in vitro in both models of ovarian cancer. In vitro, both cisplatin-resistant and cisplatin-sensitive human ovarian tumor xenograft models were used in nude mice. In experiments on mice, it was observed that quercetin liposomes significantly suppressed tumor growth in both types of models compared to normal saline, blank liposomes, and free quercetin. Thus, in the cisplatin-sensitive A2780s tumor model, the mean tumor volume of mice treated with encapsulated quercetin on day 31 was 393 ± 155 mm^3^, compared to (a) the group treated with free quercetin (1528 ± 356 mm^3^) (b) the group treated with blank liposomes (3592 ± 728 mm^3^), and (c) the group treated with NS (4171 ± 1181 mm^3^). Moreover, complete tumor regression was observed in one of the five mice that received treatment with quercetin liposomes. On the other hand, in the cisplatin-resistant A2780cp tumor model, the mean tumor volume of mice treated with encapsulated quercetin was 346 ± 178 mm^3^, compared to (a) the group treated with free quercetin (1263 ± 262 mm^3^), (b) the group treated with blank liposomes (2387 ± 491 mm^3^), and (c) the group treated with NS (3287 ± 580 mm^3^). In addition, immunofluorescence and immunohistochemistry tests determined that encapsulated quercetin inhibited tumor proliferation, induced apoptosis in both A2780cp and A2780s cell models, and reduced microvessel density [75].

Overall, quercetin liposomes could be an effective anticancer treatment for cisplatin-resistant and cisplatin-sensitive ovarian cancers [75]. Following these studies, it was concluded that, by encapsulating QC, a poorly soluble compound in water, positive results could be obtained in terms of solubility, bioavailability, and anticancer therapeutic efficacy.

### 5.2. Curcumin

**Figure 6:**
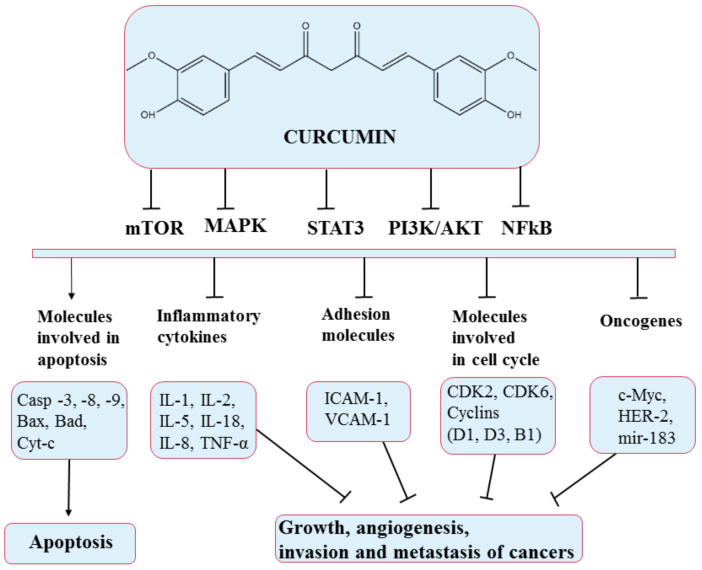
Chemical structure of curcumin and mechanism of action of curcumin through different molecular targets, resulting in apoptosis or growth suppression, angiogenesis, invasion, and metastasis of cancers.

Curcumin is a natural yellow polyphenolic compound extracted from turmeric roots (*Curcuma longa*). It is a multifunctional compound that has been widely used in traditional medicine due to its various therapeutic activities in anti-inflammation, antioxidation, antiproliferation, and anti-angiogenesis [82]. Curcumin acts on several pathways, producing growth suppression and angiogenesis, as illustrated in Figure 6.

Numerous studies have determined that curcumin has significant antitumor effects on cancer signaling pathways. It inhibits the activation of several important molecules in tumor development such as nuclear transcription factors (NF-kB), cell-cycle regulators (cyclin D1, D3, B1), cytokine mediators (IL-1, IL-8, IL-18), or enzymes (COX2). It can also induce the release of cytochrome C, as well as cause the activation of caspases 8, 3, 7, and 9 and the tumor suppressor pathway (p53). According to numerous studies [83,84], these effects lead to the death of pancreatic adenocarcinoma cells.

Nevertheless, similar to the other polyphenols, curcumin has certain drawbacks, such as low oral bioavailability and high light sensitivity. Meanwhile, its stability is unaffected by temperature, even at 250 °C. Curcumin is unstable in solution when the pH is higher than 5, and its degradation rate increases dramatically as the pH of the solution rises [85].

Researchers have attempted to find new drug delivery systems such as liposomes, solid dispersion, microemulsion, and micelles to overcome these obstacles. Among the alternatives, liposomes have been studied the most, concluding that curcumin-encapsulated liposomes have more significant growth-inhibitory and proapoptotic effects on cancer cells [85].

Curcumin liposomes have been tested on several cancers, and the effects produced by these liposomes on various types of cancers are summarized in Table 1.

As shown in Table 2, liposomes can improve curcumin’s antitumor and pharmacological activities by increasing the pharmacokinetic and pharmacodynamic effect and reducing the doses required to target the tumor. In parallel with the technological developments, curcumin liposomes can be optimized such that they will become an ideal strategy in clinical practice for the treatment of cancers [85].

### 5.3. Honokiol

Honokiol is a lignan found in several species of the genus *Magnolia*, which is distributed worldwide [91,92]. Honokiol is obtained by purification of the bark and seed cones of the magnolia tree [93]. It is known that extracts from *Magnolia* bark are used as traditional herbal medicines in Korea, China and Japan, and other countries [94]. Many lignans with anticancer potential act by shrinking the tumor, decreasing the expression of estrogen, insulin growth factor, vascular endothelial growth factor, and matrix metalloproteinases enzymes, and enhancing caspase 3 [95].

**Figure 7:**
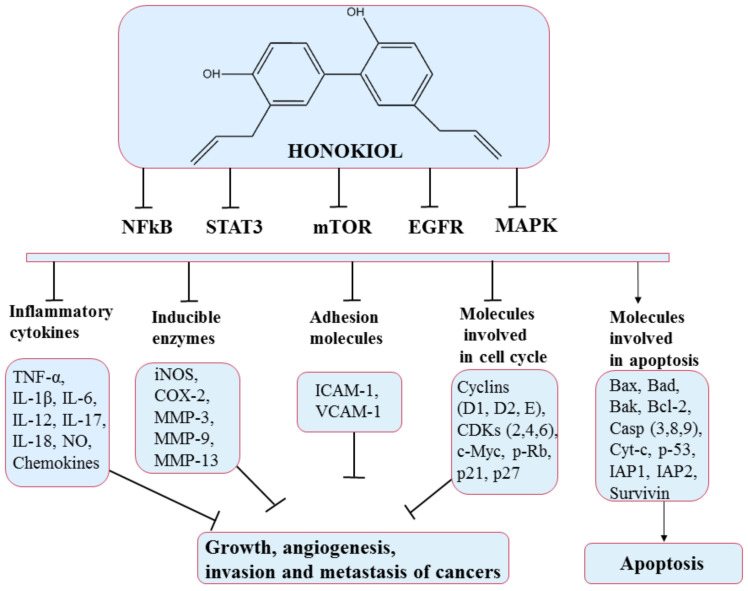
Chemical structure of honokiol and mechanism of action of honokiol through different molecular targets that suppress growth, angiogenesis, and invasion of cancers (adapted from Arora et al., 2012).

This polyphenol produces effects on many molecular targets that modulate the expression of genes controlling the different hallmarks of cancer [96]. Mechanisms of action include retarding the cell cycle (via effects on cyclins D and B) [97], inducing apoptosis (by upregulating the expression of proteins that control apoptosis such as Bcl-xL, Bcl-2), inhibiting angiogenesis (via regulation of hypoxia-inducible factor 1-alpha (HIF1α) and VEGF gene expression) [98,99], and interdicting invasion and metastasis [100]. Some of these pathways and molecules implicated are illustrated in Figure 7. Honokiol has also been shown to produce promising results in the case of chemoresistance. It shows chemosensitization effects when combined with well-known chemotherapeutics [96].

Studies performed on honokiol have found that this compound produces many pharmacological effects such as antimicrobial, antioxidation, antithrombotic, anti-inflammation, anxiolytic, and anti-atherosclerogenic activities [96,101]. Numerous studies have shown antiproliferative effects produced by honokiol on various types of cancer (brain, breast, blood, bladder, colon). The concentrations sufficient to significantly inhibit cell proliferation or cell viability of various cancer cell lines were in the range of 0–150 μM [102]. However, it is known that some chemotherapeutic agents can cause severe systemic toxicity and several undesired side effects due to their deficient pharmacokinetic characteristics and nonspecific distribution in the body [103].

Honokiol is a lipophilic compound that is insoluble in water; thus, its effectiveness is low in free form. The pharmacokinetic effects of honokiol were studied in rats. This lignan was administered intravenously, and then blood tests were performed, which indicated quick distribution and rapid decrease. Thus, the effects of this compound are reduced due to its hydrophobicity and instability [65]. These disadvantages of honokiol limit its application in clinical cancer treatment. Taking these into account, it is crucial to create new strategies to enhance the aqueous solubility and improve the bioavailability of honokiol. To overcome the shortcomings of this polyphenol, it is possible to encapsulate it in a nanocarrier specifically designed to target tumor tissues. The most effective distribution systems in the clinic are liposomes, and they can control the release of the encapsulated compound, target the tumor passive/active, and most importantly, improve aqueous solubility [104].

In addition, it should be noted that honokiol, when administered in combination with a chemotherapeutic agent, can enhance the antineoplastic effects produced on tumor cells. A study by Wang et al. (2014) showed that honokiol improved in vitro cytotoxicity of a chemotherapeutic agent, paclitaxel, against several cervical cancer cell lines. Combined treatment of the two compounds resulted in a significant increase of almost 10–60% in apoptotic cells. At the same time, cell viability was inhibited compared to treatment with honokiol alone. Furthermore, the authors reported that the average tumor size was significantly reduced compared to the control group, with shrinkage of the tumor taking place without affecting body weight. In this way, it was suggested that, under the tested conditions, the combined treatment with honokiol and paclitaxel produced low or almost zero toxicity on the normal cells [105].

Although honokiol can suppress the proliferation of cancer cells, the challenge of this compound is related to its insolubility in water, and this feature prevents the targeted administration of a concentration that will produce the desired effects at the site of the tumor [106]. Therefore, to remove this disadvantage of honokiol, many studies have been performed that included this polyphenol encapsulated in liposomes.

Luo and his team encapsulated honokiol in liposomes (Lipo-HNK) and demonstrated that they are highly effective against the cisplatin-resistant ovarian cancer cell line A2780cp. Therefore, after 21 days, a decrease in tumor volume was observed in mice treated with Lipo-HNK (408 ± 165 mm^3^), compared to mice treated with liposomes only (2575 ± 701 mm^3^) and control mice (2828 ± 796 mm^3^). At the same time, liposomes encapsulated with honokiol prolonged survival and induced intratumor apoptosis in vivo [106]. In another study, a treatment consisting of liposomes with honokiol and cisplatin was administered on murine models of colon cancer CT26. Similar results were obtained in terms of inhibition of tumor growth. This combination treatment had stronger antiproliferative effects than the treatment with encapsulated honokiol alone or cisplatin alone due to significantly higher levels of apoptosis and significantly reduced endothelial cell density [107].

In a study from 2018, cationic liposomes were modified with hyaluronic acid (HA) and encapsulated with honokiol and daunorubicin. Subsequently, the liposomes were tested against breast cancer, following their action in eliminating vascular mimicry (VM). The findings of this study were that HA-modified daunorubicin plus honokiol cationic liposomes enhanced the cellular uptake and destroyed VM channels. In addition, changes in liposomes led to prolonged blood circulation and accumulation at the target site of the tumor to produce maximum anticancer effects [64].

Overall, honokiol has generated massive interest due to its many beneficial pharmacological properties. This polyphenol has anti-inflammatory effects and has shown promising activities against multiple types of cancer. Honokiol, however, has poor aqueous solubility, which adversely affects its intestinal absorption. This disadvantage has been addressed by developing liposomal formulations. This delivery system with honokiol should consolidate its importance as a potential anticancer agent for future research [91].

### 5.4. Resveratrol

Resveratrol is a stilbenoid natural polyphenol, isolated for the first time in 1939 from *Veratrum grandiflorum* [108]. Since its first certification, resveratrol has been identified in various plants such as plums, pistachios, berries, and peanuts. However, the most abundant source is represented by fresh grape skin, where it occurs in concentrations as high as 50–100 mg/g [109,110]. In recent years, due to its beneficial effects on health, resveratrol has received the attention of researchers [111]. It is found in two isomeric forms, *cis* and *trans*, but the predominant isomer is *trans*, which has the most potent therapeutic effects due to its conformation [110,112]. In addition, it is also obtained via chemical or biotechnological synthesis from yeast *Saccharomyces cerevisiae* for industrial applications [113].

Resveratrol is sensitive to light, pH, and high temperatures due to its unstable hydroxyls and C=C double bond. In this regard, many studies have aimed to increase its stability in an effort to expand its use [114]. The *trans* form of resveratrol is stable under acidic conditions at room or body temperature, but resveratrol degrades rapidly when the pH is alkaline. Therefore, by lowering the temperature and pH and by limiting the exposure to light and oxygen, the stability of this isomer can be improved [115].

Similar to the other polyphenols, resveratrol has a low bioavailability due to poor absorption and rapid metabolism of glucuronidated and sulfated compounds, followed by their excretion [116]. Hence, the poor bioavailability of this compound is a major problem in amplifying its effects in humans and, as such, so many approaches have been attempted to increase its bioavailability [117]. The effectiveness of resveratrol depends mainly on a combination of factors such as dosage, method of administration, the origin of the targeted tumor, and other substances present in the diet that may interfere with this polyphenol [111]. Thus, to improve the bioavailability, it is necessary to carry out studies on the delivery routes, the formulations and modulation of resveratrol metabolism, and possible interactions of resveratrol with other food components. On the other hand, another possible approach to its bioavailability is creating novel resveratrol-based derivatives [118].

**Figure 8:**
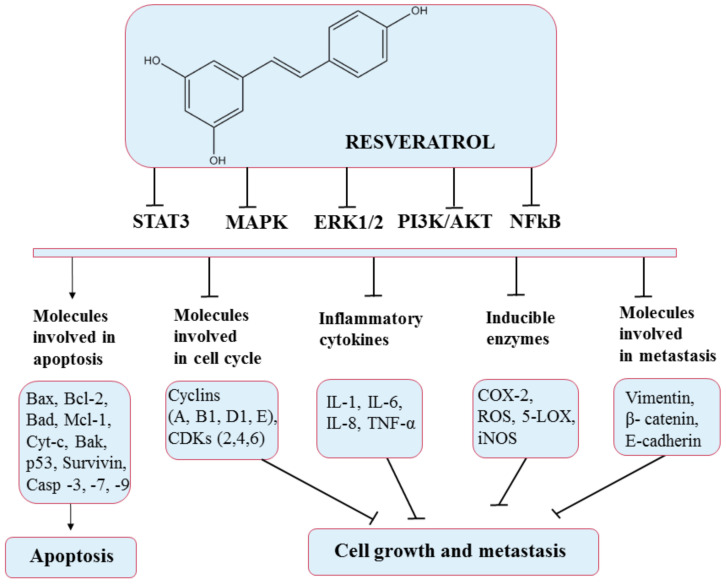
Chemical structure of resveratrol and its mechanism of action through different molecular targets that result in apoptosis or growth suppression and metastasis of cancers.

Despite the extremely low bioavailability of this polyphenol, recent studies found strong evidence that resveratrol can prevent or delay the onset of cancer, heart disease, ischemic and chemically induced damage, diabetes, pathological inflammation, and viral infections [111]. In particular, resveratrol has shown anticancer effects by altering glycolysis and molecules involved in the cell cycle (resveratrol upregulates p53 protein, thereby downregulating the expression of nuclear factor kappa-light-chain-enhancer of activated B cells (NF-κB) [119] and suppressing cancer cell growth (stops cell cycle at G1 and G1/S phases by inducing the expression of CDK inhibitors and proliferation. Resveratrol causes a high production of nitric oxide synthases (NOS), thus inhibiting cell proliferation [120], inducing apoptosis (through the intrinsic pathway via the activation of caspase 3 and caspase 9, the determined release of cytochrome c, upregulation of Bax expression, and downregulation of Bcl-2 expression [119]) promoting antitumor immune responses, and preventing cancer cell adhesion, migration (also reduced by resveratrol through the EGFR/PI3K signaling pathway [121]), and invasion by modulating active molecules and gene expression through various signaling pathways (Figure 8). In addition, different doses of resveratrol may induce different effects, which can sometimes be opposite [122]. Therefore, it is crucial to identify the most effective dose and administration route. Likewise, it has been documented that resveratrol induces cell death in tumor tissues with relatively no effect on normal tissues in the vicinity of the tumor [123]. Mukherjee et al. (2010) reported that lower doses of resveratrol could result in health benefits, while higher doses affect tumor cells via proapoptotic effects [124]. Thus, future studies based on this polyphenol are needed to fully decipher its effects.

Given both the advantages and the disadvantages of resveratrol, different methods of encapsulation and transport systems for this compound have been tried over the years to benefit to the maximum from its anticancer capabilities. One of the most used formulations is represented by liposomes, as discussed further below.

Numerous studies have investigated the efficacy and feasibility of resveratrol-loaded liposomes in targeting cancer cells. For a better distribution of liposomes to cancer cells, it is possible to modify the surface of the liposomes, adding different types of substances, including phospholipids. These added substances have the role of improving the cellular absorption of liposomes, preventing their metabolic clearance in vivo, and targeting, more specifically, the desired tissues [125].

For the first time in treating glioma tumors, Vijakumar and colleagues (2016) proposed using liposomes with resveratrol. Thus, they prepared liposomes via the thin-film hydration methodology, using phosphatidylcholine, 1,2-distearoyl-*sn*-glycero-3-phosphoethanolamine–polyethylene glycol (DSPE-PEG), and cholesterol. The liposome developed in this study showed a longer systemic circulation time and higher accumulation in brain tissue than free resveratrol, demonstrating the effectiveness of this system of targeting cancer cells in the brain [126]. Although the first study was a success, the same research team continued experiments on liposomes with resveratrol, later improving this resveratrol delivery system in the same year. In this case, the liposomes were coated with d-α-tocopherol polyethylene glycol 1000 succinate (TPGS) to increase resveratrol’s bloodstream circulation time and brain delivery. The effects of these resveratrol-loaded liposomes were evaluated both in vitro and in vivo, whereby they were used in rat glioma cells, observing significantly higher cytotoxicity for encapsulated resveratrol. In addition to cytotoxicity tests, biodistribution tests of the compound were also performed, and an increase in the amount of resveratrol present in the brain was observed, further indicating the potential of this formulation to passively target the brain [127].

Jhaveri et al. (2018) designed negatively charged resveratrol-loaded liposomes with transferrin modified on the surface (Tf-RES-Ls), through the thin-film hydration method. Transferrin molecules were added to the surface of the liposomes to enhance uptake from glioblastoma cells. The surface of the liposome can be modified to have unique specifications by adding different compositions or types of phospholipids [128]. The addition of these compounds is most often aimed at improving the cellular uptake of liposomes. Additionally, the vesicle can be protected from metabolic clearance in vivo, and the specific targeting of proteins can also be improved using this technique [129]. Likewise, in vitro experiments of competitive binding of glioblastoma cells from humans showed that the addition of the transferrin molecules enhanced cellular uptake via the transferrin receptor-mediated endocytic pathway. The authors demonstrated that liposomes with resveratrol could arrest the cell cycle and induce apoptotic cell death by activating effector caspases. This liposome (Tf-RES-Ls) demonstrated higher cytotoxicity against U-87 MG cells than the Res-liposomes in a dose-dependent manner, associated with ROS generation in tumor cells. For in vivo experiments, U-87 MG tumor xenograft bearing mice were used. The results proved that Tf-Res-liposomes significantly inhibited the tumor growth and increased the survival of mice compared to free resveratrol, showing their efficacy in the treatment of glioblastoma tumors [128]. This study concluded that these target delivery systems for resveratrol could be suitable for anticancer therapy.

Another study based on resveratrol-encapsulated liposomes was performed by Meng et al. (2016). They co-encapsulated, resveratrol and paclitaxel (PTX) in PEGylated liposomes (PEG was used to decrease the capture of liposome by the liver or spleen) and sought to determine their effectiveness for the reversal of drug resistance in breast cancer cells. To evaluate the antitumor capacity of these liposomes on drug-sensitive and drug-sensitive breast cancer cells, a standard viability assay was used, which revealed that the highest toxicity on the cell line was associated with the liposomes containing PTX and a higher dose of resveratrol. For the in vivo part of the study, nude mice with the drug-sensitive MCF-7 or the drug-resistant MCF7/Adr xenografts were used, which were given an intravenous injection of liposomes containing PTX and resveratrol. The results demonstrated that this type of liposome had a strong inhibitory effect on tumor growth and its volume, thus demonstrating that co-encapsulation of the two substrates in liposomes was efficient in the treatment of drug-resistant malignancies [130].

### 5.5. Anthocyanins

Considering their great health benefits, this section focuses on anthocyanins. Anthocyanins are pigments that are responsible for the various colors (blue, red, purple) of fruits, flowers, and vegetables. They can be stacked in vegetative tissues, where they fulfill the role of protection against biotic and abiotic factors. In nature, anthocyanins are found in the form of glycosides, which have one or more sugars bound to the aglycone nucleus. The chemical structure is C6–C3–C6, with two benzene rings (A and B) and a heterocyclic C ring. Due to this structure, anthocyanins have been shown to have strong antioxidant, anticancer, anti-inflammatory, and cardioprotective activities, as well as effects related to vision improvement [131,132].

The most frequent anthocyanin aglycones found in plants are delphinidin, cyanidin, petunidin, peonidin, pelargonidin, and malvidin. Nevertheless, these compounds are found in plants at different levels. Cyanidin has the largest proportion in plant tissues (50%), followed by pelargonidin, peonidin, and delphinidin, each representing a percentage of 12%; finally, petunidin and malvidin each make up a percentage of 7%. The unique properties of anthocyanins and anthocyanidins may have an impact on their anticancer effectiveness, antioxidant activities, and bioavailability [133].

Like other classes of polyphenols, anthocyanins can cause anti-inflammatory and antitumor effects on several types of cancer cell lines such as breast, liver, colon, prostate, ovarian, and skin cancers. These anticancer effects appear to be linked to cancer cell growth suppression via increased oxidative stress biomarkers and induction of apoptosis via the mitochondrial route [134]. As a consequence, they seem to be attractive therapeutic options.

**Figure 9:**
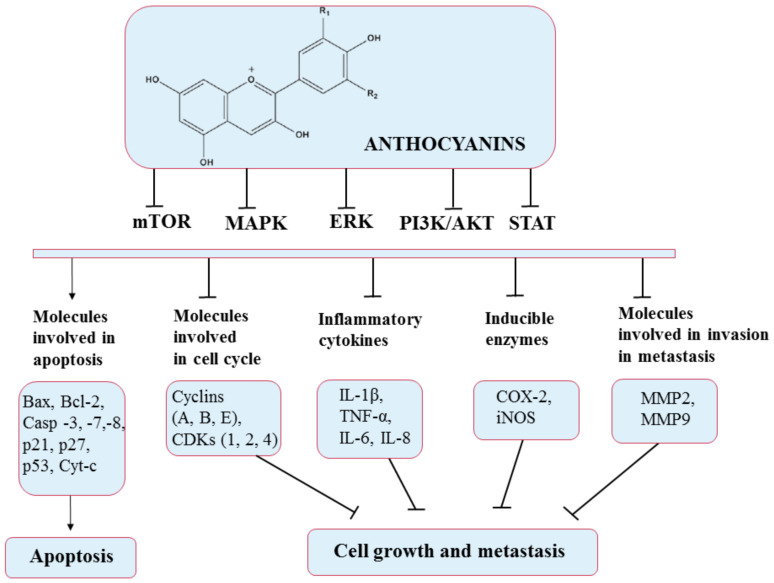
Chemical structure of anthocyanins and their mechanism of action through different molecular targets that result in apoptosis or suppressing growth and metastasis of cancers.

Anthocyanins work as antioxidants by scavenging free radicals and lowering lipid peroxidation and ROS levels. Numerous studies have also reported that anthocyanins can mediate oxidative stress in many signaling pathways such as PI3K/Akt/mTOR and Ras/ERK/MAPK, targeting their component molecules. In addition, anthocyanin-rich formulations have been found to suppress H_2_O_2_ and TNF-α-induced VEGF expression, as well as promote caspase pathways, thereby exerting anticarcinogenic and antiangiogenic effects. They also exhibit anti-inflammatory activities by significantly reducing cyclooxygenase-2 (COX-2), inflammatory interleukins (ILs), inducible nitric oxide synthase iNOS, and NF-κB. Furthermore, anthocyanins can stimulate the apoptosis of cancer cells by activating cell death receptors such as BAX and Bcl-2 and caspases 3, 7, and 8 [135]. Additionally, anthocyanin extracts were reported to mediate cell metastasis by inhibiting matrix metallopeptidase 2 (MMP-2) and matrix metallopeptidase 9 (MMP-9) through the PI3K signaling pathway [133]. All these pathways and the molecules involved are presented in Figure 9.

In recent years, intensive studies have investigated the chemopreventive potential of anthocyanins in their free form. Thus, in 2017, Wang and colleagues tested the effects of anthocyanin and anthocyanidin from a blueberry extract on the melanoma cell line B16-F10. Following the treatment of melanoma cells with the two extracts, both viability and cell proliferation were suppressed. Treatment with the anthocyanidin extract with a concentration of less than 200 μg/mL stopped the cell cycle in the G0/G1 phase, whereas, at higher concentrations, it also blocked the cell cycle in the G2/M phase. Similarly, the anthocyanin extract with a concentration of less than 400 μg/mL produced cell-cycle arrest in the G0/G1 phase. According to the findings, anthocyanin and anthocyanidin extracts might be utilized for topical and skin cancer treatments [136].

Another study tested anthocyanins from chokeberry and red grape extracts on the B16-F10 and A375 melanoma cell lines [137], reporting similar results. After treatment with both extracts, cell proliferation was inhibited, and oxidative damage was induced in cancer cells. In addition, the same extracts were tested on a normal cell line, in which case no adverse effects were monitored on healthy cells.

Although anthocyanins have been shown to have beneficial effects on cancer cells, their effectiveness is reduced due to their low stability. Along with their sensitivity to environmental factors such as pH, temperature, light, oxygen, and enzymes, anthocyanins also have a low bioavailability [138]. In addition, these polyphenols show a low absorption rate in the digestive tract. However, their encapsulation could minimize these drawbacks [131]. Several anthocyanin encapsulation systems such as nanocomplexes, nanoemulsions, nanoparticles, and liposomes have been developed to improve their stability and bioavailability [138], as further detailed below.

Different encapsulation methods, including lyophilization, spray drying, and ionic gelation, were found to have favorable impacts on anthocyanin stability in various reports. Due to these methods, liposomes can be utilized to effectively encapsulate anthocyanins.

Hwang et al. (2013) encapsulated anthocyanin from *Hibiscus sabdariffa* into liposomes to improve their stability and tested them on A375 melanoma cell line. Thus, this system significantly reduced the melanin content of melanocytes in the cell line. On the 2,2-diphenyl-1-picryl-hydrazyl-hydrate (DPPH) radical, it had an enhanced scavenging action compared to anthocyanins administered in free form. In melanoma A375 cells, there was a strong inhibition of melanin production due to decreased tyrosinase activity, as well as the expression of tyrosinase-associated proteins and melanocyte-inducing transcription factors. All these effects obtained were dependent on the dose of anthocyanins administered. Accordingly, the results of this study may underpin future melanoma treatment strategies [66].

### 5.6. Epigallocatechin-3-Gallate (EGCG)

Tea is one of the most widely consumed beverages worldwide. Green tea (*Camellia sinensis*), which is made from unfermented leaves, has been demonstrated to have the highest concentration of effective antioxidants [139]. In this type of tea are found four flavanol derivatives epicatechin (EC), epigallocatechin (EGC), epicatechin gallate (ECG), and epigallocatechin gallate (EGCG) [140]. The main polyphenolic component and the most abundant and physiologically active catechin found in green tea is epigallocatechin-3-gallate (EGCG), which is an ester of epigallocatechin and gallic acid [141,142]. EGCG is a flavone-3-ol type polyphenol, which has in its chemical structure eight free hydroxyl groups, an arrangement that makes it bioactive with versatile biological functions. In addition, aside from some tannin compounds, EGCG has the strongest free-radical-scavenging capacity among common phenolic compounds [143].

**Figure 10:**
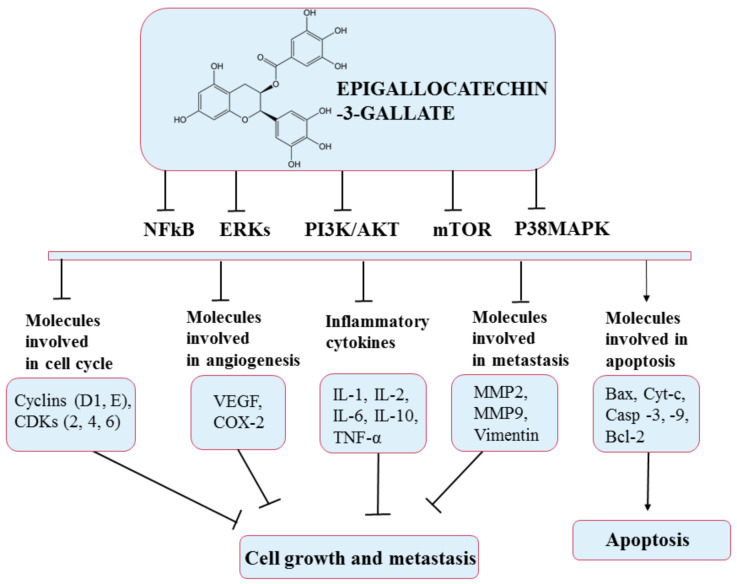
Chemical structure of epigallocatechin-3-gallate and its mechanism of action through different molecular targets that result in apoptosis or growth suppression and metastasis of cancers.

This compound has been intensively studied in recent years; research has shown its beneficial effects through its antioxidant, antibacterial, anticancer, antidiabetic, and antiangiogenic properties [142]. The chemopreventive effect of EGCG has a solid basis in studies conducted both in vivo and in vitro in a number of cancers: breast, duodenum, prostate, colon, skin, lung, cervical, and liver [140,144]. Thus, EGCG interferes with various mechanisms such as cell proliferation and differentiation, apoptosis, angiogenesis, and metastasis, with inhibitory effects on several processes in diverse types of cancer such as initiation, promotion, and progression. For example, EGCG has been shown to induce apoptosis in breast cancer by activating apoptosis-related proteins, such as caspase 3 and 9, and in cholangiocarcinoma by inducing apoptotic molecular signals, such as Bax and cytochrome c [141]. Furthermore, EGCG inhibits invasion and epithelial–mesenchymal transition through modulation of MMP-2, MMP-9, and Vimentin [145]. In addition, EGCG has been shown to stop cell division in a variety of cell lines at different stages of the cell cycle [144]. The anticancer effects of EGCG occur via several signal transmission pathways such as MAPK and PI3K/AKT, as presented in Figure 10 [146].

However, the anticancer capacity of EGCG depends on the dose, requiring a significant amount of this compound to observe its antitumor effects; this is a challenge because, in everyday life, the amount of green tea consumed by individuals is low, and the bioavailability of this compound is also weak [146]. We must also emphasize that high doses of EGCG can cause cytotoxicity in vitro, whereas, in vivo, individuals may suffer from hepatotoxicity, nephrotoxicity, and gastrointestinal disorders (vomiting and diarrhea) [147]. Although EGCG has a high activity, it cannot be easily used in the drug industry or as a natural additive due to its oxidability and instability, which compromise its bioavailability. Regardless of the mode of administration of this compound in a biological system, its bioavailability will be low [148]. Most of the ingested EGCG is absorbed in the small intestine and then degraded by microorganisms in the large intestine, leaving only 0.2–2% of the initial amount in the body [147]. As a result, it is critical to comprehend EGCG’s possible toxicity, dosages, and use.

Accordingly, one method that can improve the effects of EGCG is its incorporation into liposomes. These phospholipid vesicles can protect the molecule from degradation, thereby increasing the concentration of EGCG that reaches the target site, as well as its absorption capacity [148].

Therefore, a study by De Pace et al. (2013) concluded that EGCG encapsulated in chitosan-coated nanoliposomes had antiproliferative and proapoptotic effects in MCF7 human breast cancer cells at concentrations of 10 µM or lower, whereas free EGCG had no positive effects at such concentrations. Following this 10 µM dose of chitosan-coated liposomes, significant antiproliferative and proapoptotic effects were observed, resulting in a 40% decrease in cell proliferation and a 27% induction of apoptosis of the MCF7 cell line compared to treatment with free EGCG. Thus, EGCG encapsulated into liposomes had improved stability, sustained release, and increased intracellular concentration [149].

In a 2006 study, several cell lines of colon cancer, melanoma, and carcinoma were treated with liposomes encapsulating EGCG. The data from the study showed that encapsulated EGCG resulted in greater inhibition of cell growth in all three cell lines compared to free EGCG. The authors found that injecting liposomes into tumors was the most effective way to target cancer cells, resulting in significant EGCG accumulation in tumor tissues. In addition to generating greater EGCG accumulation inside the BCC cell line, the liposome system has the potential to function as an effective carrier of tea catechins and enhance the stability of EGCG inside the vesicles. Furthermore, encapsulated EGCG produced a higher BCC cell death effect compared to treatment with non-encapsulated EGCG at lower concentrations [60].

In a relatively recent study, a co-delivery liposomal system containing both EGCG and paclitaxel, a compound used in invasive cancer therapy, was performed. This liposome was tested using a breast cancer cell line, and it was observed that this combination produced a synergistic effect that led to the apoptosis of cancer cells and inhibition of cell invasion, processes that were deduced by increasing the activity of caspase-3 and decreasing MMP expression [150].

On the other hand, the encapsulation of EGCG in liposomal systems can inhibit the enzymatic degradation of this compound and simultaneously improve its membrane permeability. Thus, it can be stated that liposomes are good carriers to promote EGCG absorption via oral administration [151].

These results demonstrate the effectiveness of EGCG encapsulated in liposomes on various types of cancer; hence, this compound is of interest for future anticancer therapies. However, future studies are needed to understand its mechanisms of action and potential effects.

## 6. Future Perspectives and Conclusions

Although liposomes have already been used in various fields, their applicability in anticancer therapy is not fully understood. Future perspectives in the treatment of cancer using liposomes should focus on targeted delivery, considering mainly the tumor microenvironment component of each patient to efficiently target the tumor, in both preclinical and clinical studies.

In the studies done so far, polyphenols have been successfully encapsulated in liposomes, but further studies are needed in terms of encapsulation efficiency, which varies depending on the encapsulated polyphenol, the substances used to obtain liposomes, and their surface characteristics. Other existing problems with the encapsulation of polyphenols need to be solved such as the incomplete degradation of the transporter in the body (liposome), nonspecific accumulation in organs, and lower drug loading. Additionally, studies are necessary to analyze the chemical stability of substances after their incorporation into liposomes and how the components of vesicles influence the activity of polyphenols to determine potential anticancer effects.

Another argument in support of the need for further studies is that the evidence for the beneficial effects of polyphenols is not very convincing because most of the data obtained originate from in vitro experiments or animal models, where compounds were used at doses higher than people can assimilate daily into their diet. Thus, to obtain high concentrations of polyphenols in the body, they must be encapsulated and specifically targeted.

The most current strategy for using encapsulated polyphenols in the treatment of cancer involves the use of a combination of liposomes with polyphenols and chemotherapies or radiotherapies, thus producing a much stronger synergetic effect. In the same direction, in order to obtain more substantial anticancer effects, different bioactive substances have been co-encapsulated with the purpose of producing synergistic effects. However, multiple studies must be performed to understand the mechanisms of action of these compounds, how synergy occurs, and which cell types will have the desired effects.

Lastly, with the improvement of liposome technologies, it would be helpful to develop a protocol that can be used on a large scale while reducing the manufacturing costs of liposomes, which are currently relatively high. In addition, the evolution of technology will determine the emergence of other new nanomaterials that can be used in the manufacture of new drug delivery systems or in the improvement of existing ones, thus offering the opportunity for wider clinical use of polyphenols in the treatment of cancer.

## Figures and Tables

**Table 1:** Liposomal formulations that have been created for polyphenol-related biological research.

Polyphenol	Production Method	% (*w*/*w*) Polyphenol/ Lipids	Encapsulation Efficiency	Biological Effects	Ref.
**Curcumin**	Lyophilization (Freeze-drying) Evaporation method with some modification Thin-film hydration Ethanol injection	10–25 15 N/S N/S	45% ± 0.2% 73.7% ± 1.6% 87.8% ± 4.3% 46.6% ± 1.0%	In vivo: antiangiogenic activity and tumor growth inhibition Enhanced stability Slower release and better accumulation More stable during storage	[46,47] [48] [49] [50]
**Resveratrol**	Lyophilization (Freeze-drying) Thin-film hydration Film hydration	20 10 N/S	N/S >90% 78.14% ± 8.04%	Prostate cancer incidence was minimized, and bioavailability was enhanced The toxicity of free resveratrol was considerably lowered Enhanced delivery	[51] [52] [53]
**Quercetin**	Film hydration and lyophilization procedure Film hydration and sonication Emulsification/evaporation	30 N/S 10	N/S 87.1% ± 2.7% 69.42–85.72%	Enhanced solubility, bioavailability, and antitumor activity in vivo Maintained higher plasma quercetin concentrations Inhibited growth of glioma cancer cells	[24] [54] [55]
**Silymarin**	Film hydration Reverse evaporation technique Supercritical fluid technology	20 10 N/S	92.56% ± 0.93% 69.22% ± 0.6% 91.4%	Better oral bioavailability Higher bioavailability Enhanced oral bioavailability	[56] [57] [58]
**Dehydro-** **silymarin**	Film hydration and freeze-drying	25	81.59% ± 0.24%	Better oral bioavailability	[59]
**Epigallocatechin-3-gallate (EGCG)**	Film hydration and sonication/extrusion Film hydration Reverse-phase evaporation method	20 10 N/S	84.6% ± 3.8% 80% ± 3% 85.79% ± 1.65%	Protection against deterioration Even at lower doses, there was an increase in carcinoma cell death Enhanced targeted delivery and controlled release Modulated the proliferation of tumor cells	[60] [61] [62]
**Fisetin**	Film hydration and extrusion Probe sonication	18 7–15	58% N/S	Enhanced bioavailability and antitumor activity Better antiangiogenic and anticancer activities	[23] [63]
**Honokiol**	Film hydration and sonication Film hydration	20 N/S	95.43% ± 2.76% 90.1% ± 2.3%	Strong anticancer effect on breast cancer Enhanced cytotoxicity and cellular uptake Enhanced bioavailability and promoted accumulation in tumor	[64] [65]
**Anthocyanins**	Film hydration Hydration and ultrasound combined Improved supercritical carbon dioxide (SC-CO_2_)	N/S 4.5–9 20	43% 50.6%	Enhanced antioxidant activity Enhanced chemical stability and bioavailability Enhanced stability and bioavailability	[66] [67] [68]

**Table 2:** Types of cancers and the effects of curcumin treatment encapsulated in liposomes.

Cancer Type	Cell Line	Effects	IC_50_ Free	IC_50_ Encaps	Ref
**Lung cancer**	Liposomes modified with polyethylene glycol + polyethylenimine, curcumin-loaded, tested on A549 cells	Cell delivery was optimized. Stronger anti-cancer activities	30.0 ± 9.5 μM	1.4 ± 0.1 μM	[86]
**Cervical cancer**	Liposomes coated with carboxymethyl dextran, curcumin-loaded, tested on Hela cells	Optimized stability and better cell delivery, longer retention period and leak protection Increased cytotoxicity	24.8 μM	6.6 μM	[87]
**Breast cancer**	Curcumin-loaded nanoliposomes, tested on MCF-7 cells	Cell-cycle arrest was suppressed depending on the dose administered, and apoptosis occurred Bioavailability was improved	20 ± 1.8 μg/mL	11.5 ± 1.1 μg/mL	[88]
**Osteosarcoma**	Curcumin-loaded γ-cyclodextrin liposomes tested on KHOS cells	Increased cytotoxicity activities Better uptake	22.8 ± 1.9 μg/mL	6.4 ± 0.7 μg/mL	[89]
**Liver cancer**	Cationic liposomes curcumin-loaded, tested on HepG2 cells	Increased cytotoxicity activities	30 µM	4 µM	[90]

## Data Availability

Data sharing not applicable.

## Abbreviations

EGCG	Epigallocatechin-3-gallate
QC	Quercetin
CDK2	Cyclin-dependent kinase 2
EMT	Epithelial–mesenchymal transition
PEG	Polyethylene glycol
ROS	Reactive oxygen species
HIF1α	Hypoxia-inducible factor 1-alpha
Lipo-HNK	Honokiol in liposomes
HA	Hyaluronic acid
VM	Vascular mimicry
NF-κB	Nuclear factor kappa-light-chain-enhancer of activated B cells
NOS	Nitric oxide synthases
DSPE-PEG	1,2-distearoyl-*sn*-glycero-3-phosphoethanolamine–polyethylene glycol
TPGS	d-α-Tocopherol polyethylene glycol 1000 succinate
Tf-RES-Ls	Resveratrol-loaded liposomes with transferrin modified on the surface
PTX	Paclitaxel
TNF-α	Tumor necrosis factor
COX-2	Cyclooxygenase-2
iNOS	Inducible nitric oxide synthase
MMP-2	Matrix metallopeptidase 2
MMP-9	Matrix metallopeptidase 9
DPPH	2,2-Diphenyl-1-picryl-hydrazyl-hydrate
EC	Epicatechin
EGC	Epigallocatechin
ECG	Epicatechin gallate
